# Public health round-up

**DOI:** 10.2471/BLT.25.011225

**Published:** 2025-12-01

**World toilet day**

Safe and climate-resilient sanitation systems are fundamental for health, dignity and development. Yet 3.4 billion people still lack access to a safe toilet. Poor water, sanitation, and hygiene contribute to 1.4 million deaths annually. On world toilet day, the World Health Organization called global leaders for urgent action: investing in future-ready toilets and ensuring safe sanitation for all.

**Figure:**
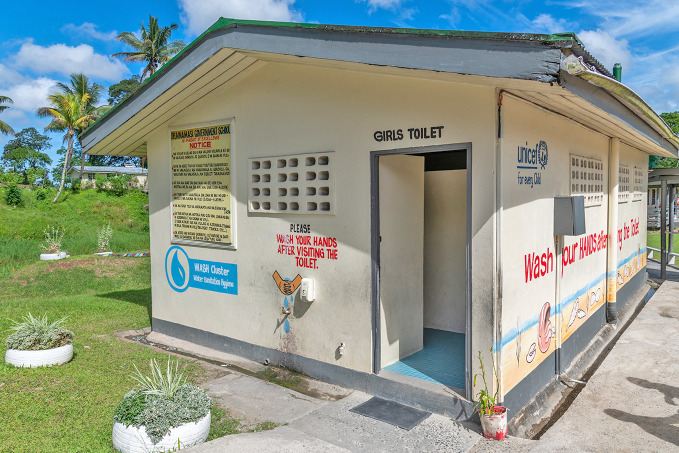
WHO/Tom Vierus

## Violence against women persists

Violence against women remains one of the world’s most persistent and under-addressed human rights and public health crises, with almost no progress in two decades, according to a new report from the World Health Organization (WHO) and United Nations (UN) partners.

Nearly one in three women, around 840 million globally, have experienced intimate partner or sexual violence in their lifetime, a figure largely unchanged since 2000. In the past year alone, 316 million women were subjected to physical or sexual violence by a partner. Non-partner sexual violence also affects millions, with 263 million women reporting such abuse, though experts warn this is heavily under-reported.

"Violence against women is one of humanity’s oldest and most pervasive injustices, yet still one of the least acted upon," said WHO Director-General Tedros Adhanom Ghebreyesus. "No society can call itself fair, safe or healthy while half its population lives in fear."

The report calls for urgent investment in prevention, survivor-centred services and stronger laws, warning that funding for these efforts is collapsing despite rising risks.

https://bit.ly/49XSRLe

## Health and climate

Climate change is already driving a global health crisis, with over 540 000 deaths from extreme heat each year and one in 12 hospitals worldwide at risk of climate-related shutdowns, according to a new report released by WHO and Brazil’s 30^th^ Conference of the Parties (COP30) Presidency.

The *COP30 Special report on health and climate change: delivering the Belém health action plan* highlights mounting health impacts as global temperatures already exceed 1.5°C above pre-industrial levels. Currently, 3.3 to 3.6 billion people live in areas highly vulnerable to climate change, and hospitals face a 41% higher risk of damage from extreme weather compared to 1990. 

Without rapid decarbonization, the number of health facilities at risk could double by mid-century. The report calls for urgent investment in resilient health systems, integration of health objectives into climate plans and community-led adaptation strategies. It warns that only 54% of national health plans assess risks to facilities, leaving critical gaps.

The report follows the launch of the Belém Health Action Plan, a flagship initiative of Brazil’s COP 30 Presidency, unveiled on the dedicated Health Day of COP30 – 13 November 2025.

“The report provides clear data and evidence that climate change is already directly affecting health systems around the world,” said Dr Alexandre Padilha, Minister of Health, Brazil. “The Belém Health Action Plan and this report offer countries the tools they need to turn scientific evidence into concrete action.”

https://bit.ly/4ogyhte

## Kangaroo mother care guide

WHO marked its first official world prematurity day on 15 November, by releasing a new global clinical practice guide for kangaroo mother care (KMC), a simple, proven intervention that saves lives and improves outcomes for preterm and low birth weight babies.

Each year, 15 million babies are born too soon, and complications from preterm birth remain the leading cause of death among children under five years of age. In low-income countries, most extremely preterm babies die within days, while in high-income countries, nearly all survive. KMC, prolonged skin-to-skin contact combined with breast-milk feeding, reduces newborn deaths by over 30%, hypothermia by nearly 70%, and severe infections by 15%, while improving growth and long-term development.

The new WHO guide provides step-by-step instructions for initiating and maintaining KMC in all health settings and at home, emphasizing family involvement and supportive policies.

“KMC is not just a clinical intervention – it empowers mothers and families and transforms newborn care,” said Jeremy Farrar, WHO Assistant Director-General for Health Promotion, Disease Prevention and Care. “It should now be universal clinical practice for all small and preterm babies, ensuring they have the best chance to survive and thrive.” 

https://bit.ly/4plmB9s

## Drug-resistant gonorrhoea

WHO warns that gonorrhoea, a common sexually transmitted infection (STI), is becoming increasingly resistant to antibiotics, according to new data from its enhanced gonococcal antimicrobial surveillance programme (EGASP). The findings underscore the urgent need for stronger surveillance, better diagnostics and equitable access to new treatments.

Between 2022 and 2024, resistance to ceftriaxone, the main antibiotic for gonorrhoea, rose from 0.8% to 5%, while resistance to cefixime jumped from 1.7% to 11%. Ciprofloxacin resistance reached 95% and azithromycin remained stable at 4%. Cambodia and Viet Nam reported the highest resistance rates. In 2024, 12 countries across five WHO regions submitted data, up from four in 2022, signalling progress in global monitoring.

“This global effort is essential to tracking, preventing, and responding to drug-resistant gonorrhoea and to protecting public health worldwide,” said Tereza Kasaeva, director of WHO’s Department for HIV, TB, Hepatitis and STIs. “WHO calls on all countries to address the rising levels of sexually transmitted infections and integrate gonorrhoea surveillance into national STI programmes.”

https://bit.ly/3K3lqfM

## First cervical cancer elimination day

Countries and partners worldwide are marking the first-ever world cervical cancer elimination day on 17 November, a milestone agreed by Member States at the 78^th^ World Health Assembly to accelerate efforts against a preventable disease. Cervical cancer, the fourth most common cancer in women, claims over 350 000 lives annually, yet it can be eliminated through proven interventions.

The day reinforces WHO’s global strategy built on three pillars: vaccinating 90% of girls against human papillomavirus (HPV), screening 70% of women, and treating 90% of those with pre-cancer or invasive cancer. It aims to strengthen advocacy, expand services, and mobilize resources to ensure universal access to life-saving care.

Momentum is growing. Gavi, the Vaccine Alliance and partners announced that the goal to reach 86 million girls with HPV vaccination by the end of 2025 has been met. Countries are scaling up action: Sierra Leone and Liberia are launching campaigns targeting 1.5 million girls, while Malaysia promotes self-sampling HPV testing to boost screening.

World cervical cancer elimination day signals a historic opportunity to turn commitment into action and move closer to a future free of cervical cancer.

https://bit.ly/48m3GFz

## Diabetes in pregnancy 

On world diabetes day, 14 November, WHO launched its first global guidelines for managing diabetes during pregnancy, a condition affecting one in six pregnancies globally. The recommendations aim to prevent serious complications for mothers and babies and reduce long-term health risks.

Diabetes in pregnancy, if unmanaged, increases the risk of pre-eclampsia, stillbirth, and birth injuries. It also raises lifetime risks of type 2 diabetes and cardiometabolic diseases for both mother and child. The burden is greatest in low- and middle-income countries, where access to specialized care remains limited.

The guidelines outline 27 evidence-based recommendations, including individualized advice on diet and physical activity, regular blood glucose monitoring, tailored medication regimens for type 1, type 2 and gestational diabetes, and multidisciplinary support for women with pre-existing diabetes. They emphasize integrating diabetes care into routine antenatal services and ensuring equitable access to essential medicines and technologies.

“These guidelines are grounded in the realities of women’s lives and health needs, and provide clear, evidence-based strategies to deliver high-quality care for every woman, everywhere.” said Tedros Adhanom Ghebreyesus, WHO Director-General.

https://bit.ly/4i9WOyA

## Research priorities for paediatric clinical trials

WHO has published a new technical report, *The future of paediatric clinical trials – setting research priorities for child health*, outlining a global research agenda to address critical evidence gaps for children aged 0–9 years of age. Despite major advances in child health, preventable illnesses still cause significant morbidity and mortality, particularly in low- and middle-income countries. Children remain under-represented in clinical trials, leaving gaps in evidence for policies and care.

Developed through an inclusive process involving over 380 stakeholders, the agenda identifies 172 priority research questions across infectious diseases, noncommunicable diseases, newborn health, early childhood development and nutrition. It emphasizes feasibility, scalability and equitable impact, aiming to guide coordinated action and investment.

“This research agenda offers governments, partners and research institutions a clear direction for investment. By identifying where evidence is most needed, it creates an opportunity to coordinate resources and foster collaboration to address the highest-burden areas affecting children today,” said Meg Doherty, director of WHO’s Department of Science for Health.

WHO calls for strategic funding, regional collaboration, and integration of research into health systems to accelerate progress.

https://bit.ly/4pifbDA

**Cover photo**

Hoang Van Tuan, from Cao Bang province, cleans his rice fields after heavy floods caused by a series of typhoons that swept through northern and central Viet Nam during September and October 2025.

**Figure:**
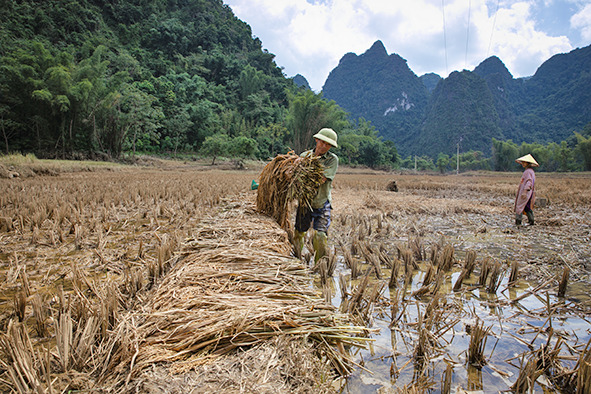
UNICEF/UNI886275/Vu Le Hoang

**Looking ahead**

1 December. World AIDS Day 2025. https://bit.ly/4a09oOE

6 December. Universal Health Coverage (UHC) High-level Forum. Tokyo, Japan. https://bit.ly/49t62nk

12 December. Launch of WHO Learning on TAP. Lyon, France https://bit.ly/49sr9Gd

